# Inverse analysis of surrounding rock parameters of loess tunnels and numerical simulation analysis of stress-seepage coupling under water migration

**DOI:** 10.1038/s41598-025-02602-x

**Published:** 2025-05-21

**Authors:** Jun-jie Xuan, Ming Li, Yao-hui Du, Jia-qi Lin, Yue Gao, Yun-cheng Mao, Kun Zhang

**Affiliations:** 1 School of Civil Engineering, Northwest Minzu University, Lanzhou, 730030 China; 2 School of Civil Engineering, Lanzhou Jiaotong University, Lanzhou, 730070 China

**Keywords:** Water-rich loess tunnels, Stress-seepage coupling, Water migration, Pore water pressure, Numerical simulation, Environmental sciences, Engineering

## Abstract

In this study, the Tuanjie Tunnel project on the Tongwei-Dingxi Expressway is utilized to investigate the stress-seepage coupling in loess tunnels. Field monitoring, laboratory experiments, and numerical simulations were employed to establish a coupled numerical model of the stress-seepage field for the shallow-buried sections of these tunnels. The seepage-stress interactions in loess tunnels were analyzed, revealing variations in pore water pressure around the tunnel and the deformation behavior of surrounding rock during construction, with particular attention to the effects of water migration.The results indicate that when the groundwater level is 10 m from the tunnel crown, the pressure of pore water at various measurement points follows an order of tunnel invert > arch springing > arch waist > arch haunch > tunnel crown. Within the pipe roof reinforcement zone, pore water pressure increases with distance from the tunnel perimeter, while above the zone, it decreases with distance. When considering water migration, the excavation of the upper bench significantly impacts the vertical effective stress at each point, the excavation of the middle bench impacts the arch wall and the haunch, and the excavation of the lower bench impacts the springing of the arch.Based on these insights, addressing the challenges encountered during the construction of water-rich loess tunnels, the implementation of pipe roof reinforcement measures for surrounding rock has played a positive role in enhancing the stability of loess tunnels during construction.

## Introduction

Numerous significant engineering disasters, attributed to water seepage-induced deformation, have been recorded in renowned civil engineering projects worldwide. Prominent examples include the tragic failure of the Malpasset Arch Dam^1^ in France in 1958 and the water inrush incident that occurred in the Shijingshan Tunnel^2^ in China in 2021. These incidents stemmed from alterations in the seepage field of surface or groundwater within the project areas, triggered by external factors, which subsequently impacted the stress states of engineering structures and surrounding rocks.

Under water-rich conditions, the migration of water has a significant impact on the stability of surrounding rocks in underground engineering. Previous research has firmly established that water considerably deteriorates the mechanical characteristics of rocks, including strength and stiffness, and consequently undermines the capacity of surrounding rocks to bear loads^3–5^. Additionally, water migration alters the pore water pressure distribution within the surrounding rocks, enhancing the risk of deformation^6–8^, and potentially modifies their physical properties by increasing fissures and porosity, further diminishing the overall strength and bearing capacity of the rocks^9,10^. Furthermore, in water-saturated environments, water infiltration can lead to internal structural damage of the surrounding rocks, accelerating weathering and degradation processes^11–13^, triggering microstructural impairments and particle loss within the rocks, ultimately weakening their stability^14–16^. Water migration may also induce chemical corrosion of rocks, provoking swelling and disintegration, which further undermines the stability of the surrounding rocks^17–19^. In a water-rich environment, the steel bars in the tunnel support structure are susceptible to water erosion. After the steel bars corrode, they will expand in volume, which in turn causes cracks and damage to the concrete structure under stress. This is also one of the main factors that lead to cracking of the primary support structure and damage to the secondary lining^20,21^.In the case of certain types of weak strata, such as loess formations, their extreme instability upon encountering water can precipitate tunnel face collapses and excessive tunnel deformation^22–24^. During the construction of loess tunnels, excavation and support measures induce alterations in the groundwater seepage field within the tunnel area, impacting the strength, water content, specific yield, and permeability coefficient of the surrounding rocks, consequently influencing their stress and deformation state^25,26^. Therefore, a profound investigation into the stability of underground structures within the coupled seepage-stress field under water-rich conditions is imperative. Such research advances a deeper understanding of the performance of underground engineering in complex hydrogeological settings and provides a scientific groundwork for design and construction.

In recent years, scholars from domestic and international arenas have undertaken extensive research on the seepage-stress coupling field and reaped substantial and fruitful achievements. Yang et al.^27^, through triaxial laboratory tests, investigated the effect of rainfall intensity and duration on the stability of shallowly buried large-section loess tunnels post-excavation, concluding that the increased water content of loess subsequent to rainfall significantly impacts the stress of supporting structures. Su et al.^28^ studied the stability and control measures for its control in water-rich tunnels, revealing that shallowly buried water-rich tunnels are prone to catastrophic failure, with advanced deformation of surrounding rock being the primary cause of large deformations. Yuan et al.^29^examined red-bed soft rock tunnels from perspectives including burial depth, lateral pressure asymmetry, and groundwater level fluctuations, comprehensively analyzing and summarizing the mechanical effects on surrounding rock deformation and support structures under stress-seepage coupling. Weng et al.^30^ dissected the seepage-damage coupling mechanism of tunnel linings under high water pressure conditions, proposing a seepage-damage coupling model capable of predicting the condition of damage of linings subjected to high water pressure. Richards^31^ extended Darcy’s Law to the investigation of unsaturated seepage problems. Sun et al.^32^ utilized a custom-designed experimental setup, observed and analyzed the seepage behavior of rock samples under varying stress conditions, elucidating the impacts of stress level, loading paths, and rock’s physical properties on seepage characteristics. Aristotelis^33^ established a extensive, heterogeneous, instantaneous, and unsaturated flow model in soil, accounting for finite flow domains and the non-stationarity of soil properties and flow characteristics. JAR Cordero^34^ devised a reliable approach by employing the finite element method to investigate the mechanisms underlying the activation of natural fractures and the ensuing process of hydraulic fracture propagation. Pedro^35^ tackled the issue of a fluid-propelled fracture expanding within a permeable poroelastic medium and formulated a zero-thickness finite element for fracture modelling. In this model, the fracture propagation is regulated by a cohesive zone model, while the fluid flow inside the fracture is dictated by the lubrication equation.Luo et al.^36^ introduced a seepage-stress coupling model employing the extended finite element method, tailored for the coupling of displacement in oil and gas reservoirs. This approach facilitates the simplification of the process of converting fluid pressures within complex fracture networks into equivalent nodal forces. Zhang et al.^37^ conducted stress-seepage coupling experiments on arenite under high stress and varying lithostatic pressures using GCTS technology, the obtained stress–strain curves effectively characterized the mechanical behavior of arenite, providing valuable insights into its mechanical properties under extreme conditions.

The research on the stress and deformation characteristics of tunnel surrounding rock during groundwater migration, alongside the accurate simulation of the stress-seepage coupling relationship within the rock matrix under the influence of water migration, assumes paramount significance. This study, leveraging the Tuanjie Tunnel project of the Tongwei-Dingxi Expressway as a case study, employs a multifaceted approach that integrates in-situ monitoring with three-dimensional numerical simulations. We establish a coupled numerical model for the seepage-stress fields in the surrounding rock of loess tunnels. Specifically, targeting the stability issues of water-rich, shallowly buried loess tunnels under seepage conditions, a finite element model is constructed for saturated loess tunnels, considering the synergistic effects of seepage and stress fields within the surrounding rock. Numerical simulations are then conducted under various operational scenarios, allowing us to derive the configuration patterns of pore water pressure, displacement field, and stress field within the surrounding rock of loess tunnels, both with and without accounting for the implications of water migration. Furthermore, the mechanical characteristic of support structures under these conditions is analyzed, providing insights into their load-bearing characteristics. This comprehensive analysis serves as a cornerstone for designing more effective reinforcement strategies and optimizing tunnel performance in the context of water-sensitive geological formations.

### Project overview

#### Project background^38^

**Fig. 1:**
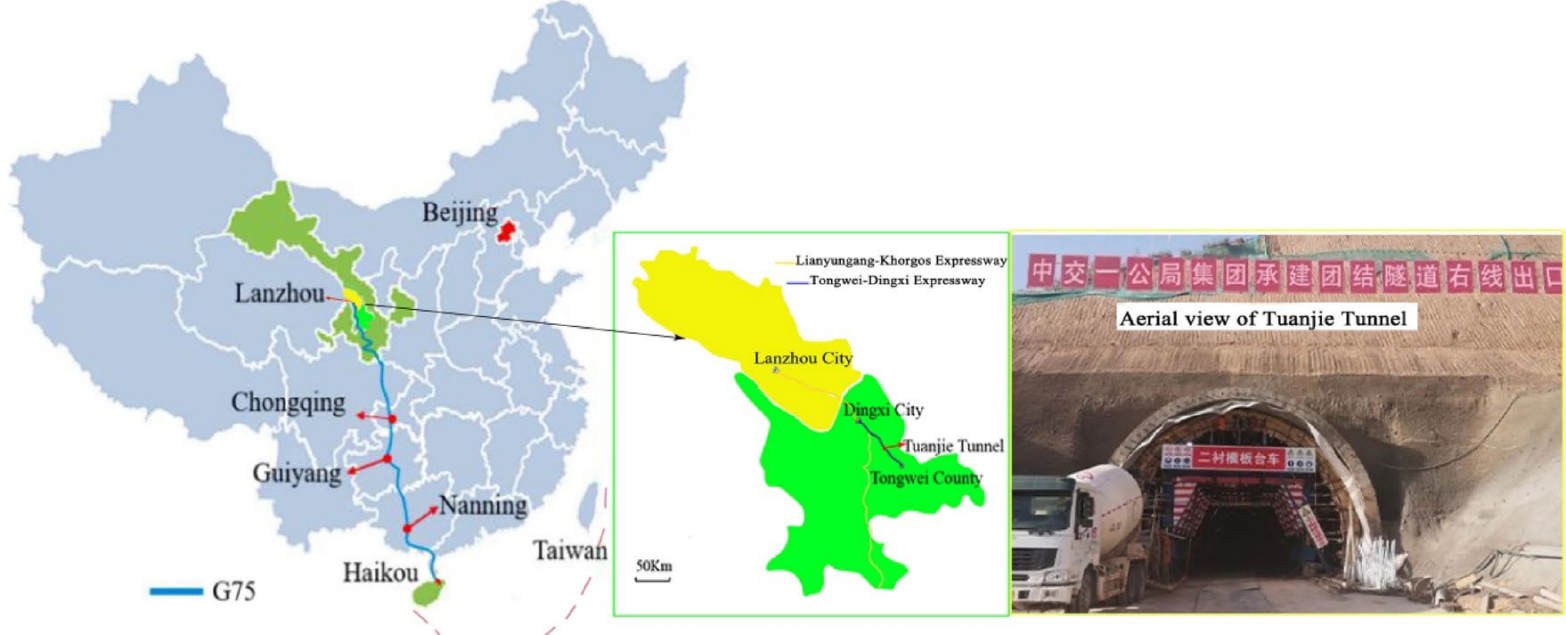
Location map and aerial view of the Tuanjie Tunnel site. The simple line drawings of the map were created based on the map (GS(2019)No.1818) issued by the Ministry of Natural Resources of the People’s Republic of China.

**Fig. 2:**
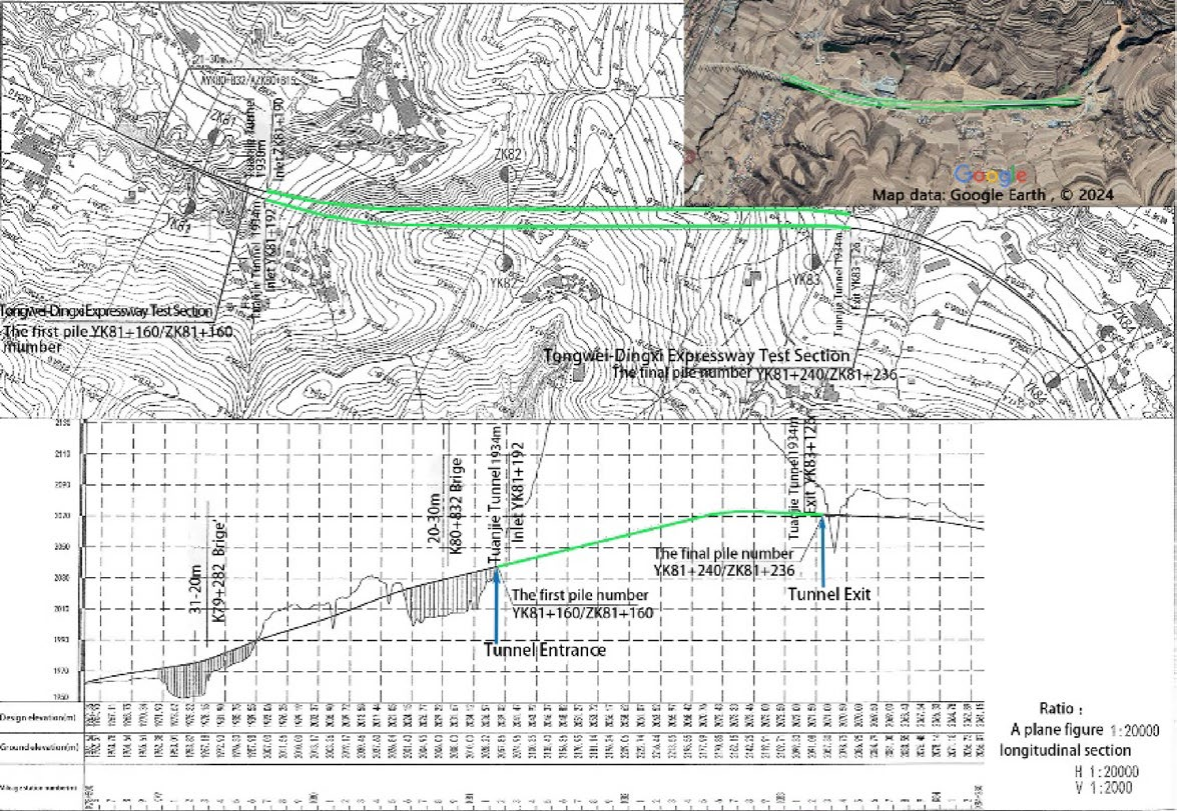
Plan and profile views of the Tuanjie Tunnel. The satellite imagery in Fig. 2 was sourced from the online website of “Google Earth”. The version number is “10.67.0.5”.

**Fig. 3:**
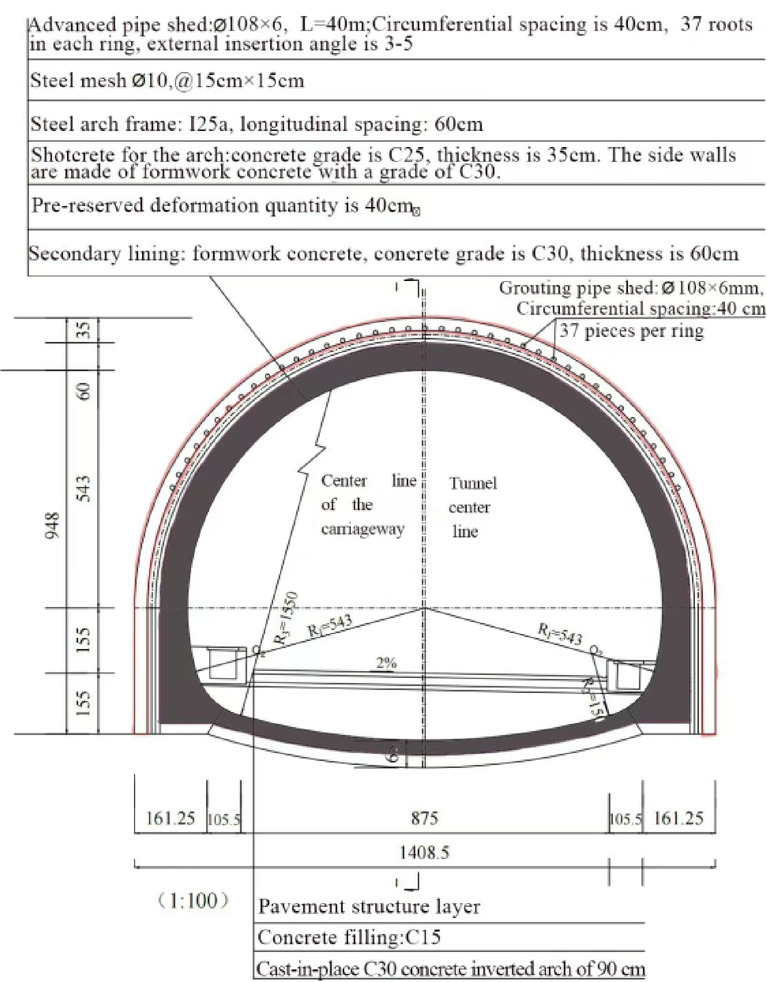
Lining design for the cross-section of the Class VI surrounding rock segment.

**Fig. 4:**
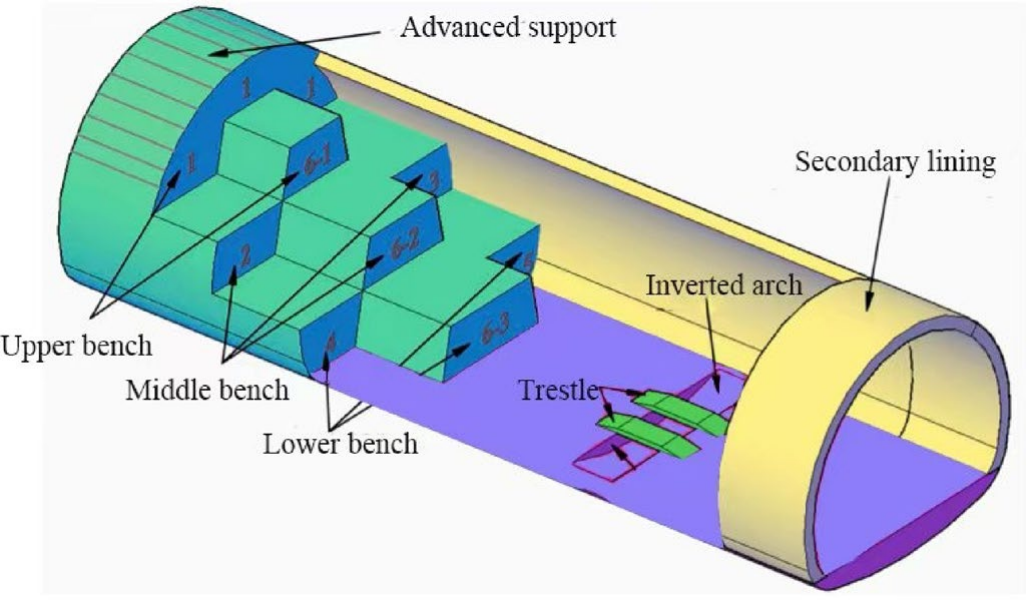
Schematic diagram of the three-bench and seven-step excavation method.

The Tuanjie Tunnel is situated atop a loess ridge to the south of Tuanjie Town, Dingxi City, Gansu Province, traversing from east to west. The right tunnel extends from milepost YK81 + 192 to YK83 + 124, spanning a total length amounts to 1,932 m, while the left tunnel ranges from milepost ZK81 + 190 to ZK83 + 236, covering a total distance of 2,046 m. The tunnel site experiences an elevational range from a maximum altitude of 2,220 m to a minimum of 2,020 m, resulting in a relative height difference of 200 m. Both the exit and entrance of the tunnel are located on loess slopes with gradients ranging from 6° to 15°, where the slope aspects intersect the tunnel alignment at a large angle. The geographical location and the construction site map of the tunnel are illustrated in Fig. 1. Additionally, the plan and profile views of the tunnel are presented in Fig. 2. In the case of the shallowly buried part of the tunnel that is characterized by Class VI surrounding rock, the tunnel cross-section boasts a height of 9.48 m and a span of 14.08 m. The detailed support parameters of the cross-sectional structure are depicted in Fig. 3. The excavation sequence of the tunnel, which employs a three-bench, seven-step excavation method, is illustrated in Fig. 4.

#### Stratigraphic lithology and hydrogeological conditions

The strata at the tunnel site are classified in terms of age and genesis, with the Quaternary soil layers being predominant, particularly the late Neogene inland piedmont-lacustrine facies and the widely distributed mid-to-upper Pleistocene loess. This comprises Holocene colluvial and deluvial loess-like soil (Q_4_^c+dl^), alluvial-proluvial silty clay (Q_4_^al+pl^), late Pleistocene eolian loess (Q_3_^eol^), and alluvial-proluvial loess (Q_3_^al+pl^). The groundwater within the tunnel site area primarily occurs within the loess layers, distributed mainly in the loess ridge area and higher terraces. The pore-phreatic aquifer medium within the loess fractures comprises mid-to-late Pleistocene eolian loess and loess-like silt, with the underlying silty clay layer acting as a relative aquitard. The primary recharge source for the pore water in loess fractures is atmospheric precipitation, with an average groundwater level of 2048.7 m. The groundwater levels at the tunnel entrance section are proximal to the tunnel crown, and these levels exhibit significant seasonal variations.

### In-site monitoring and laboratory experiments

#### In-site monitoring plan

**Fig. 5:**
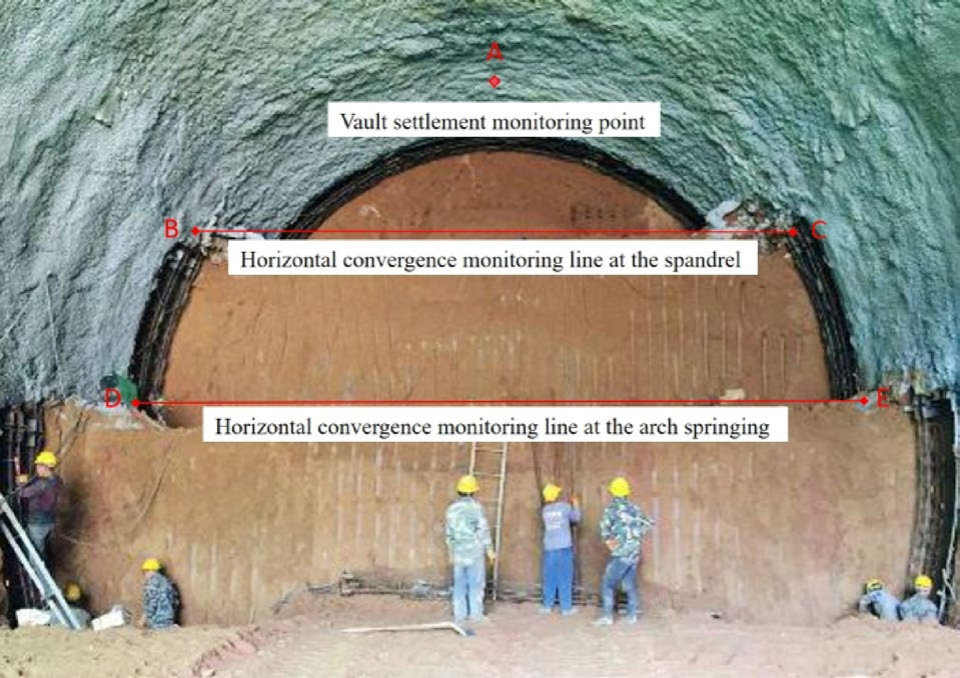
Arrangement of monitoring points for crown settlement and horizontal convergence.

**Fig. 6:**
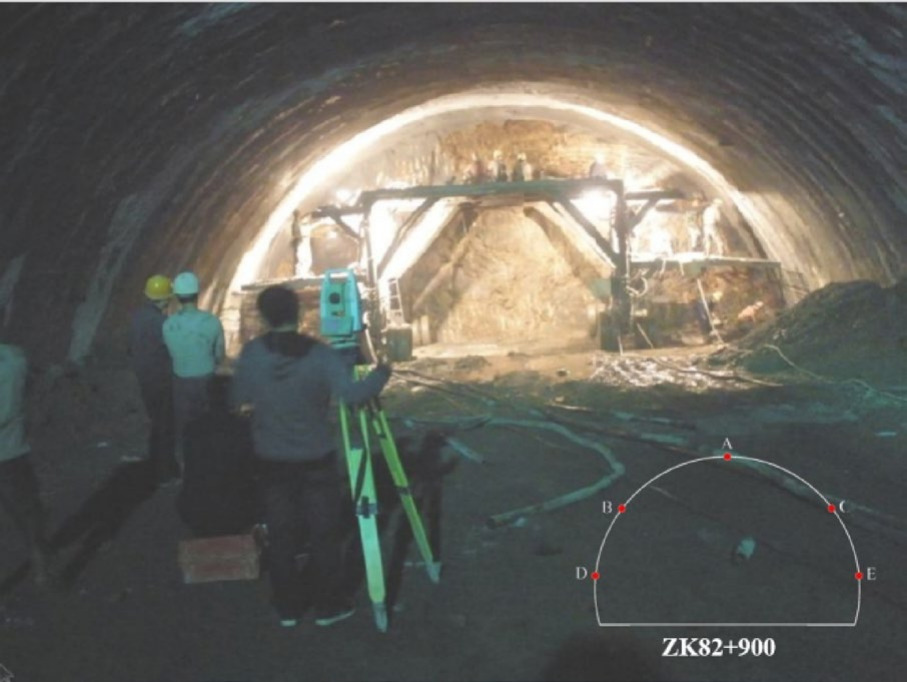
Monitoring setup at the tunnel site.

The monitoring items in this study encompass the horizontal convergence and vertical settlement of the tunnel, the contact pressure between the primary support and the surrounding rock, the stress of the steel arch frame, and the pore water pressure at various points. The selected study section is at the mileage of ZK82 + 900, with the surrounding rock type being plastic to soft-plastic loess, classified as Grade VI, with a tunnel burial depth of 46.5 m and the groundwater table within a range of 20 m from the design elevation. Due to space limitations, the following text only presents the monitoring data and pattern analysis of the surrounding rock deformation, and the layout of the displacement measurement points and the in-site monitoring situation diagram are shown in Figs. 5 and 6.

#### On-site monitoring plan

The monitoring plan was formulated with comprehensive considerations of the on-site construction scheme, construction progress, engineering geological conditions, etc. The monitoring work must closely follow the on-site construction progress, and the sensors should be installed promptly after excavation. To ensure the accuracy of subsequent data, the initial values of each instrument were first recorded using the VW-102E frequency output instrument after the sensors were installed. After the completion of the secondary lining construction of the tunnel, the GDA1602 (IV) full-function acquisition module was employed for subsequent data acquisition. The monitoring sensors, monitoring items, and monitoring frequencies used in the monitoring are presented in Table 1 below.

**Table 1:** Mechanical parameters of the surrounding rock as determined through laboratory testing.

Serial number	Monitoring items	Sensor name	Monitoring frequency		
			1 ~ 15d	16d ~ 1mo	1 ~ 3mo
1	Crown settlement and horizontal convergence	Total station and convergence meter	1 ~ 2times/d	1times/2d	1 ~ 2times/wk
2	Surrounding rock pressure	Earth pressure cell	1 ~ 2times/d	1times/3d	1 ~ 2times/wk
3	Surface stress and strain of steel arch support	Surface strain gauge	1 ~ 2times/d	1times/3d	1 ~ 2times/wk
4	Reinforcement stress of secondary lining	Rebar strain gauge	1 ~ 2times/d	1times/3d	1 ~ 2times/wk
5	Concrete stress	Concrete strain gauge	1 ~ 2times/d	1times/3d	1 ~ 2times/wk
6	Pore water pressure	Pore water pressure gauge	1time/d	1time/3d	1time/wk

#### Deformation patterns of surrounding rock at monitored cross-sections

**Fig. 7:**
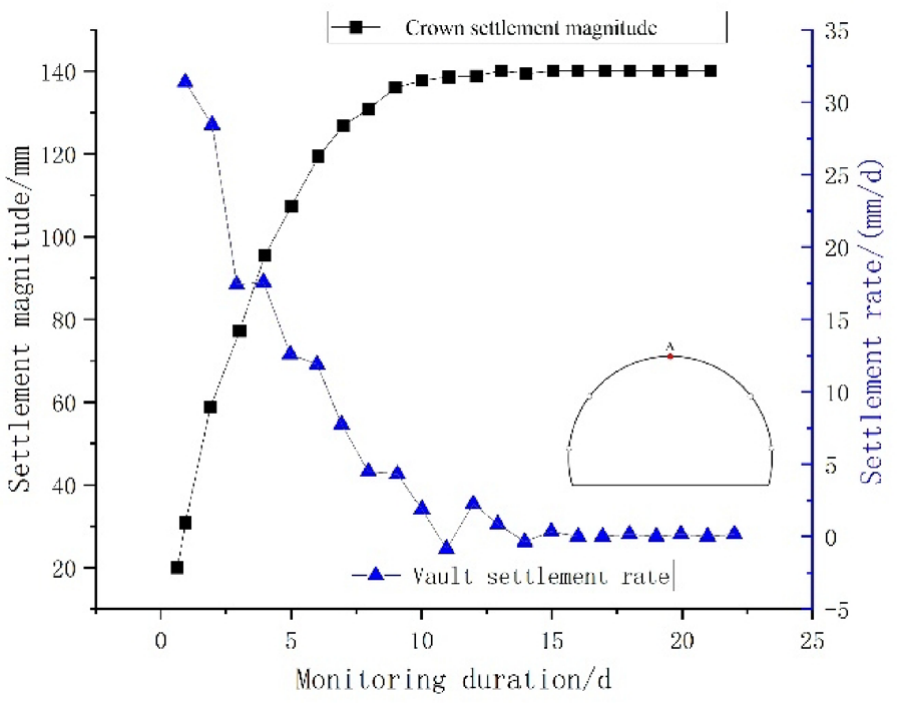
Time-history curves of arch crown settlement and settlement rate.

**Fig. 8:**
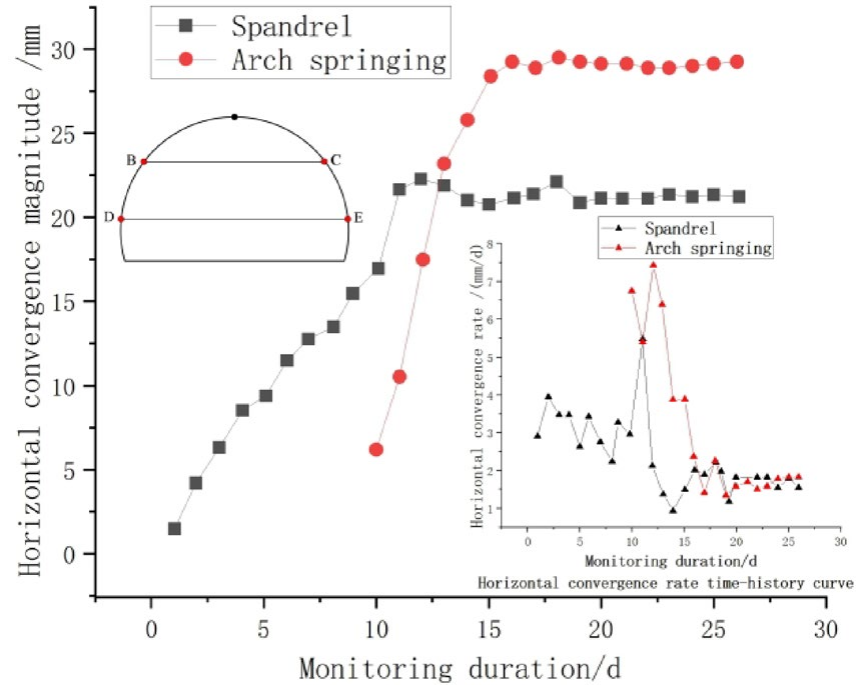
Time-history curves of horizontal convergence and convergence rate.

Figure 7 illustrates the time-history curves of arch crown settlement and settlement rate for the typical section at ZK82 + 900. Figure 8 presents the time-history curves of horizontal convergence displacement and rate for the surrounding rock.

**Fig. 9:**
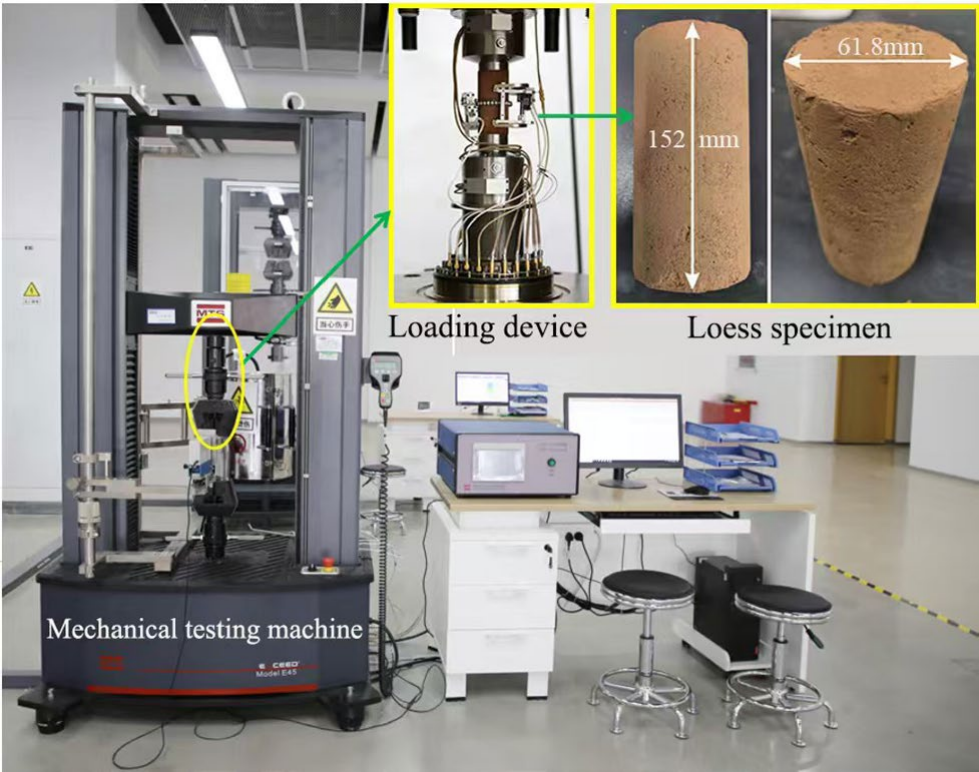
MTS mechanical experiment system.

As depicted in Fig. 9, within nine days following the excavation of the tunnel face, approximately 96.8% of the total deformation was attributed to crown settlement. Between days 10 and 12, the settlement of the vault made up roughly 1.5% of the entire deformation. Beyond day 15, the crown subsidence exhibited minimal variation and tended towards stability, with a maximum subsidence of 140.1 mm. The rate of vault settlement increased rapidly within the first five days, peaking at 31.3 mm/d, and subsequently decreasing to 0 mm/d by day 14.

**Fig. 10:**
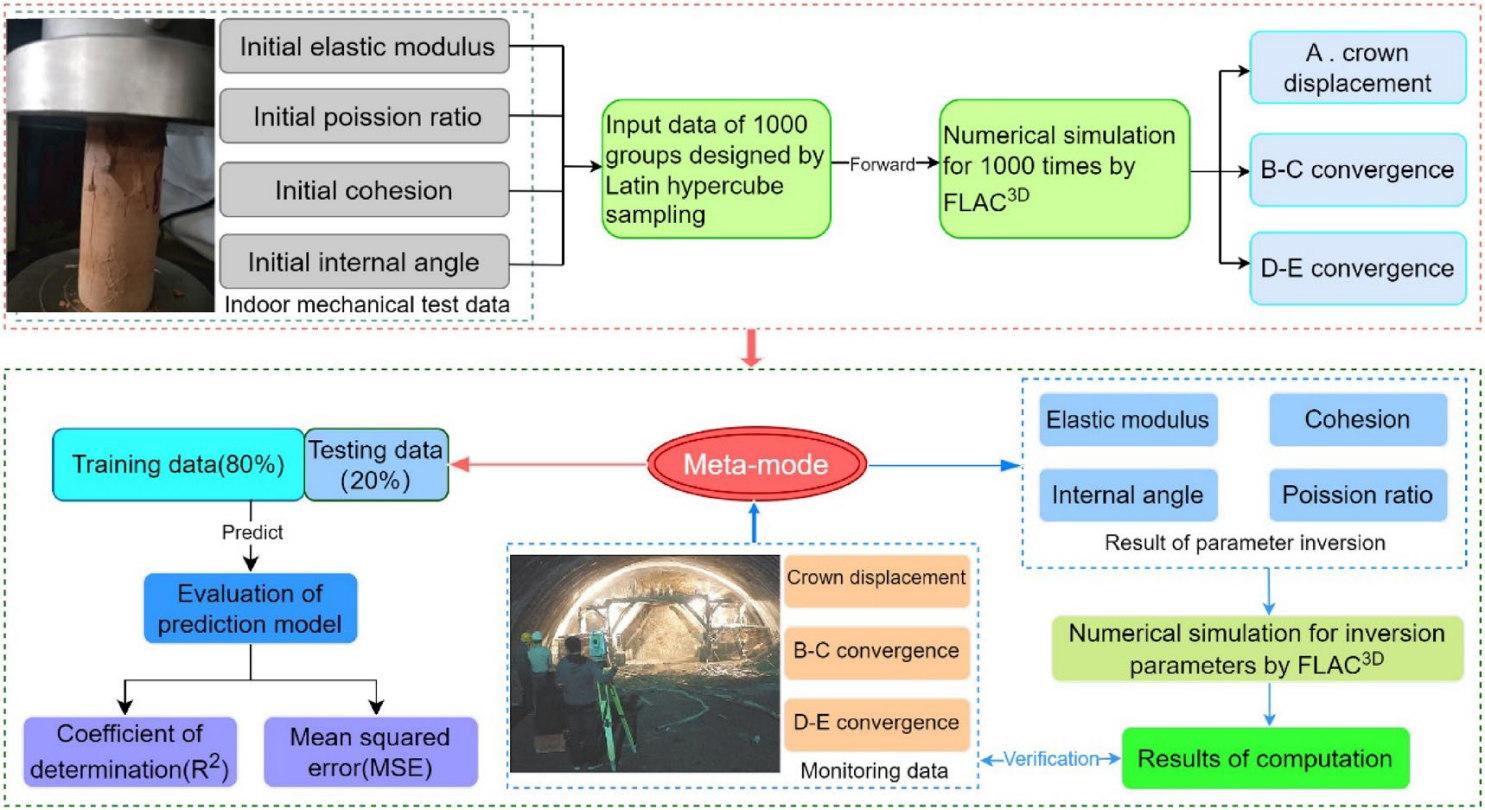
Flowchart of the parameter inversion process.

Figure 10 presents the displacement and velocity time-history curves of horizontal convergence at the monitoring section ZK82 + 900. As depicted in the figure, the horizontal displacement at the arch shoulder (line BC) exhibited rapid deformation development within 9 days after the excavation of the tunnel face, with the maximum rate of increase reaching 2.76 mm/d. On the 10th day , due to the excavation of the arch waist, the rate of increase reached a peak of 4.65 mm/d. By the 15th day post-excavation, the convergence tended to stabilize, with a value of 21.47 mm and a settlement rate of 0.1 mm/d. The horizontal convergence at the arch foot (line DE) reached its maximum value within 7 days post-excavation, which was 29.7 mm. The rate of horizontal convergence reached its maximum on the 3rd day post-excavation, with a value of 6.93 mm/d, and then gradually stabilized.

#### Laboratory experiments

The surrounding rock specimens were retrieved from the tunnel site and subsequently processed in the laboratory using a core drilling rig and a rock-cutting machine into cylindrical samples with a height of approximately 152 mm and a diameter of 61.8 mm. The relevant mechanical parameters of the rock specimens were obtained using an MTS mechanical testing apparatus^39,40^, as illustrated in Fig. 9. Upon organizing the experimental data, the initial mechanical parameters of the rock specimens were determined, as presented in Table 2.

**Table 2:** Laboratory mechanical parameters of surrounding rock.

Material	Modulus of elasticity/MPa	Specific gravity/(kN/m^3^)	Poisson’s ratio/–	Cohesion /kPa	Internal friction angle/°	Permeability coefficient/(m/s)
Aeolian loess	53.1	17.3	0.33	27.0	26.0	6.63E−07
Soft plastic loess	41.4	20.2	0.34	16.2	24.4	1.20E−07
Alluvial loess	49.6	20.3	0.36	25.8	23.9	1.98E−07

#### Theory, methodology, and process for parameter inversion of surrounding rock

In this paper, the inversion parameters are expressed as:

1 $$X = \left[ {E,\mu ,c,\varphi ,k} \right]$$

where $E$ is the elastic modulus; $c$ is the cohesion; $\mu$ is the Poisson’s ratio; $k$ is the permeability coefficient.

Based on field monitoring data, the convergence at the BC line, the convergence along the DE survey line, and the settlement value at the arch crown are represented as follows:

2 $$^{{G* = \left\{ {g_{1}^{*} ,g_{2}^{*} ,g_{3}^{*} } \right\}}}$$

where $g_{1}^{*}$, $g_{2}^{*}$, $g_{3}^{*}$ denotes the respective in-site monitoring values for BC convergence, DE convergence, and arch crown settlement.

In the simulation of tunnel excavation, the arch crown settlement at point A (*G*), the convergence along the BC survey line, and the convergence along the DE survey line are functions of the mechanical parameter (*X*), represented as:

3 $$G = f(E,\mu ,c,\varphi ,k)$$

Given a set of parameters *X* to be determined, the calculated settlements at point A (G), and the convergences at the BC and DE lines are obtained through numerical computation, expressed as:

4 $$G = \left\{ {g_{1} ,g_{2} ,g_{3} } \right\}$$

Utilizing rock mechanics experiments, engineering geological survey reports, and relevant specifications, the range of values for the parameters to be inversed is determined. The settlement of the arch crown at point A and the convergences along lines BC and DE for diverse combinations are calculated, the aim is to minimize the sum of absolute deviations from field monitoring values^41^. The expression is given by:

5 $$\Phi \left( X \right) = \sum\limits_{i = 1}^{3} {\left| {g_{i} - g_{i}^{*} } \right|}$$

In the context of numerical simulation, when the mechanical parameters reach their minimum value, they are considered to have converged toward the actual mechanical properties of the rock.

During the inversion process, A finite element numerical model pertaining to tunnel excavation was constructed.The excavation process encompasses advancement and the primary support; the parameter inversion methodology includes the validation of the meta-model, comparison of algorithms, and the verification of the forward process using the inverted parameters^42^. The overall procedure for the inversion parameters is depicted in Fig. 10 below.

#### Verification of inversion results

**Fig. 11:**
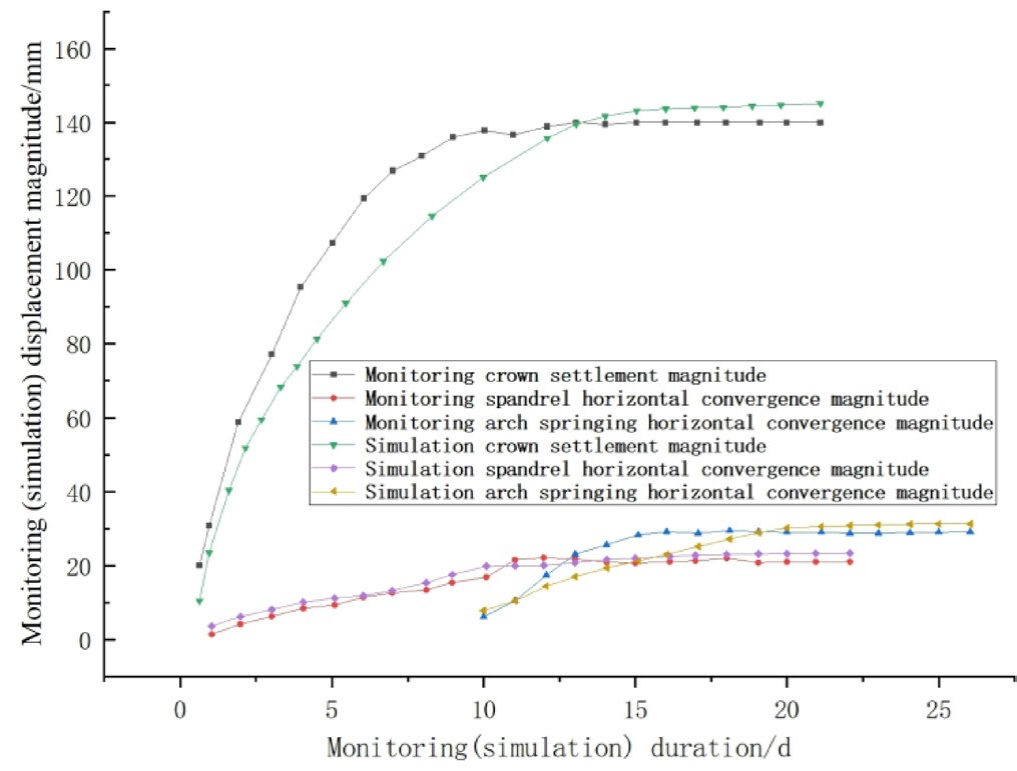
Comparison of displacement trend between monitoring data and numerical simulation data.

Three representative cross-sections (ZK82 + 890, ZK82 + 900, and ZK82 + 950) were selected for parameter inversion. The surrounding rock deformation at these sections stabilized approximately 25–30 days after excavation and support. Consequently, displacement monitoring data within 25–30 days were adopted as the objective function for parameter inversion. Based on field monitoring data—including BC survey line convergence (21.5 mm), DE survey line convergence (29.7 mm), and crown settlement at Point A (140.1 mm)—the mechanical parameters of the surrounding rock were inversely analyzed using FLAC^3D^ software (V9.0, https://www.itasca.cc/cpzx), integrating the proposed inversion methodology. The results demonstrated that the elastic modulus of Aeolian loess along the tunnel axis ranged from 52.6 to 53.8 MPa, exhibiting a strong correlation with the loess layer thickness distribution in the geological profile (R^2^ = 0.86). The maximum discrepancy in inversion results across the three sections was less than 6%, indicating remarkable consistency of rock parameters along the tunnel alignment. This consistency validates the engineering rationality of the inversion outcomes and confirms their alignment with site-specific geological conditions.The mechanical parameters of the surrounding rock obtained from the mechanical experiment using the MTS testing system mentioned in the previous text are used as the initial parameters for simulation calculations. After multiple calculations and adjustments, the inverted values of the mechanical parameters are finally obtained^43,44^, as shown in Table 3. The contrast between the measured displacement values and the numerical simulation values is shown in Table 4, and the comparison chart of their change trends is shown in Fig. 11.

**Table 3:** Back-analysis values of surrounding rock parameters.

Material	Modulus of elasticity/MPa	Specific gravity/(kN/m^3^)	Poisson’s ratio/–	Cohesion/kPa	Internal friction angle/°	Permeability coefficient/(m/s)
Aeolian loess	53.2	18.3	0.34	26.8	25.9	6.55E−07
Soft plastic loess	40.6	20.4	0.35	16.2	24.2	1.10E−07
Alluvial loess	49.1	20.2	0.38	25.7	23.9	1.91E−07

**Table 4:** Comparison between the measured displacement values and the simulation values.

Type of result	The settlement value of the crown at point A/mm	BC line convergence value/mm	DE line convergence value/mm
Measured value	140.1	21.5	29.7
Simulated value	145.3	22.4	30.8
Relative difference in error (%)	3.8	4.2	3.7

Through the comparison, it is observed that the numerically simulated values of the surrounding rock displacement are in excellent accordance with the in-situ measured values. This indicates that the mechanical parameters of the surrounding rock, derived from numerical simulation inversion, closely match the actual parameters of the in-situ surrounding rock. The data curve of numerical simulation exhibit a high degree of accuracy, thereby lending a considerable level of credibility and reliability to the use of numerical simulation methods for the analysis of the construction process.

### Numerical simulation study of loess tunnel underwater migration

#### Numerical simulation of governing equations

In finite element software MIDAS GTS NX (2023R1, https://www.midasit.cn/), pore pressure(*p*) is utilized as a variable in seepage analysis. Moreover, by deriving Darcy’s law, the control Eq. (6) for seepage analysis containing pore pressure can be obtained^45^.

6 $$\frac{1}{{\gamma_{w} }}\nabla^{T} \left( {{\mathbf{k}}\nabla p} \right) - \nabla^{T} \left( {{\mathbf{kn}}_{g} } \right) = \left( {\frac{nS}{{\rho_{w} }}\frac{{\partial \rho_{w} }}{\partial p} + n\frac{\partial S}{{\partial p}}} \right)\frac{\partial p}{{\partial t}}$$

Using the variational method on the governing equation yields the following integral formula (7^46^) :

7 $$\int_{\Omega } {\frac{1}{{\gamma_{w} }}\nabla^{T} \left( {{\mathbf{k}}\nabla p} \right)} d\Omega - \int_{\Omega } {\nabla^{T} \left( {{\mathbf{kn}}_{g} } \right)} d\Omega + \int_{\partial q} {q_{ext} } dS = \int_{\Omega } {\left( {\frac{nS}{{\rho_{w} }}\frac{{\partial \rho_{w} }}{\partial p} + n\frac{\partial S}{{\partial p}}} \right)\frac{\partial p}{{\partial t}}} d\Omega$$

In Eq. (7), *q*_*ext*_ represents the flow velocity on the surface of the model.

The interpolation of pore pressure using shape functions results in nonlinear coupled equations with respect to time, incorporating pore pressure, as presented in Eq. (8).

8 $${\mathbf{C}}\left( {P_{i} } \right){\mathbf{P}} + {\mathbf{K}}\left( {P_{i} } \right){\mathbf{P}} = {\mathbf{R}}\left( {q_{ext} ,{\mathbf{n}}_{g} } \right)$$

#### Boundary and initial conditions of the model

1. Seepage boundary conditions: Boundary conditions are applied to the nodes above the water table within the analysis domain. The software possesses the capability to automatically eliminate the boundary conditions for nodes in regions above the water table^47^.
2. Pore water pressure/initial conditions

There are two methods for defining pore water pressure: The first method involves establishing the groundwater table for saturated or unsaturated soil through boundary lines or interfaces. Secondly, it can be obtained by computing the inner product of the nodal pore water pressure p_i_ and the shape function N_i_ at the corresponding position^48^.

#### Establishment of tunnel modeling

In this study, based on the geological survey report of the tunnel, the shallow-buried section at the exit (ZK82 + 900 ~ ZK82 + 930) was selected as the subject of numerical analysis. The tunnel burial depth within the studied section ranges from 20 to 50 m, with the groundwater table fluctuating within a range of 10 m above the tunnel crest.

**Fig. 12:**
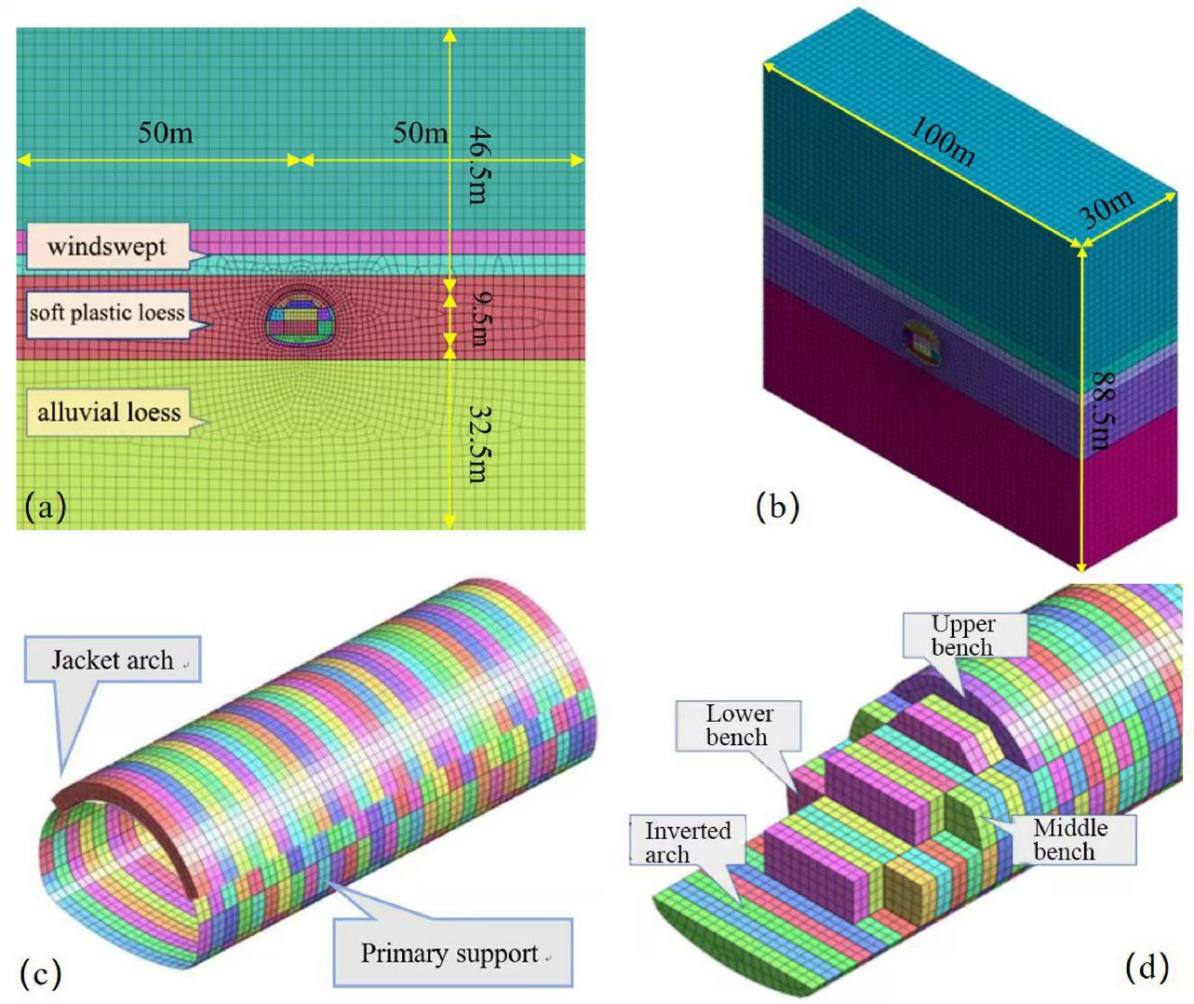
Diagram of the numerical simulation calculation model.

According to the principle of Saint–Venant, the upper dimension of the model is set to 46.5 m based on the burial depth of the tunnel, with the lower boundary 32.5 m away from the base of the tunnel arch; the boundaries are each 50 m from the central axis of the tunnel, as shown in the frontal view of the model in Fig. 12a. The determination of the model’s depth primarily depends on the construction safety distance for the formation of the initial support loop. Based on the actual conditions at the construction site, this model adopts a depth of 30 m, making the entire model a box-shaped entity measuring 100 m in length, 30 m in depth, and 88.5 m in height, as shown in the overall model diagram in Fig. 12b. The primary support and jacket arch reinforcement of the model are depicted in the form of arch units in Fig. 12c. In conjunction with the actual in-site project, the construction steps are selected using the micro-stage seven-step method for tunnel excavation. The tunnel excavation is divided into four excavation platforms: upper, middle, lower, and inverted arch, with seven working faces, staggered to excavate simultaneously and advance step by step, as shown in Fig. 12d.

To ensure the accuracy of the established numerical model and its similarity to the actual situation on site as much as possible, the mesh size of the excavation area, the area within 3 m of the excavation range, and the pipe-roof reinforcement area is controlled to be 0.5 m. The mesh size of the surrounding rock at a relatively long distance from the tunnel is controlled to be 1 m. The entire model utilizes a hybrid mesh generator for automatic mesh generation. The pipe-roof reinforcement area, the secondary lining, and the surrounding rock are simulated by solid elements, and the meshes of the solid elements are hybrid hexahedral meshes. The primary support is simulated by plate elements, which are obtained through solid extraction. The locking anchor pipes are simulated by implanted truss units.

#### Model calculation parameters

The soil material parameters utilized in the simulation, as well as the mechanical indices of the support structures, are determined based on an integrated assessment of the geological survey data, design drawings, and specification requirements of the Tuanjie Tunnel, establishing the relevant mechanical parameters of the stratum. The stratum is modeled using solid elements. The mechanical parameters of the soil and various support materials are presented in Table 5.

**Table 5:** Mechanical parameters of surrounding rock and support structures for the tunnel.

Material	Modulus of elasticity/MPa	Specific gravity/(kN/m^3^)	Poisson’s ratio/–	Cohesion/kPa	Internal friction angle/°	Permeability coefficient/(m/s)
Aeolian loess	53.2	18.3	0.34	26.8	25.9	6.55E−07
Soft plastic loess	40.6	20.4	0.35	16.2	24.2	1.10E−07
Alluvial loess	49.1	20.2	0.38	25.7	23.9	1.91E−07
Pipe arch reinforcement	1.3	23.0	0.25	200.0	45.0	1.32E−08
Primary support	26.0	22.0	0.20	–	–	–
Secondary Lining	30.0	25.0	0.20	–	–	–
Steel rib	210.0	78.5	0.20	–	–	–
Preceding pipe arch	210.0	50.6	0.25	–	–	–

#### Basic assumptions of the model

1. Boundary condition settings

The displacement boundary conditions for the entire model are as follows: the vertical displacement at the base is set to zero; the horizontal displacements on both sides are constrained to be zero, with free vertical movement; the ground surface is free boundary conditions. For the seepage boundary conditions, only the seepage under the influence of gravity is considered; during the self-weight stress equilibrium step, all boundaries are impermeable. During the excavation process, the inner boundary of the lining and the units at the excavation face are set with zero pore water pressure boundaries; all other boundaries are considered non-draining^49^.

(2) Assumptions for model calculations

The initial stress field is derived from the self-weight stress field; the soil calculation model is a Mohr–Coulomb elastoplastic model, while the lining is modeled using a linear elastic model; Assuming that pre-support measures (e.g., forepoling and steel mesh reinforcement) are regarded as safety reserves, the primary support system is considered to rely solely on the reinforcing effects of steel arches and shotcrete; It is supposed that the pore water pressure is constant before the tunnel is excavated, and the seepage adheres to Darcy’s law^50^.

(3) Realization of the construction process

The implementation commenced with the application of soil self-weight, followed by a self-weight stress equilibrium calculation to establish the initial in-situ stress field prior to tunnel excavation. This resultant stress state served as the initial condition for subsequent excavation simulations. Subsequently, the passivation and reactivation functions of the software were employed to simulate the lining elements during construction phases. By modifying their material properties, the supporting effect was achieved. Finally, the groundwater migration process was simulated through the adjustment of seepage boundary conditions.

## Results

### Coupled stress-seepage analysis of surrounding rock considering water migration

#### Pore water pressure analysis

**Fig. 13:**
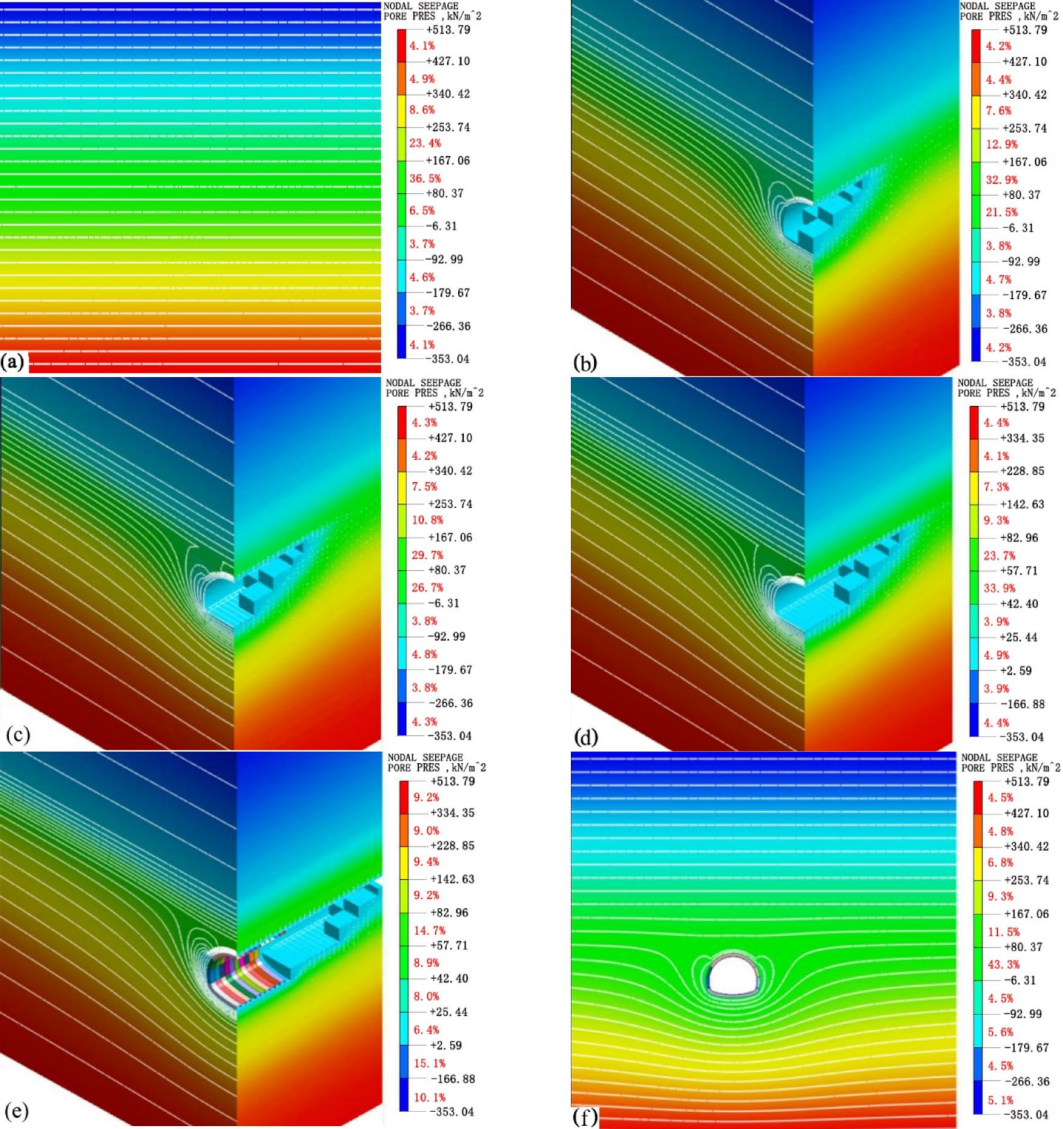
The contour maps of pore water pressure at each stage of underwater migration: (**a**) Initial stage; (**b**) Excavation of the upper bench; (**c**) Excavation of the middle bench; (**d**) Excavation of the lower bench; (**e**) Excavation of the inverted arch; (**f**) Completion of excavation.

Figure 13 below presents the pore water pressure nephograms at each excavation stage considering water migration.From the figure, it can be seen that before tunnel excavation, the pore water pressure of the surrounding rock is horizontally stratified along the vertical direction, as shown in Fig. 13a. With the progressive excavation of the upper bench, middle bench, and lower bench, the pore water pressure around the tunnel gradually decreases. The isograms of pore water pressure in the horizontal direction show a decreasing trend, and the overall distribution gradually takes on a funnel-like shape, as shown in Fig. 13b–d. After the invert excavation is completed, the isograms of pore water pressure at the arch bottom are denser than those in other parts, as shown in Fig. 13e. After all the excavations are completed, for various locations around the tunnel, the pressure head on the inner side of the tunnel rapidly becomes 0, causing a sharp change in the distribution curve of the pressure head in the seepage field. The isograms of pore water pressure at the crown and spandrel are significantly sparser than those at the arch bottom. The further away from the tunnel perimeter, the more stable the variation trend of the pore water pressure becomes, as shown in Fig. 13f.

**Fig. 14:**
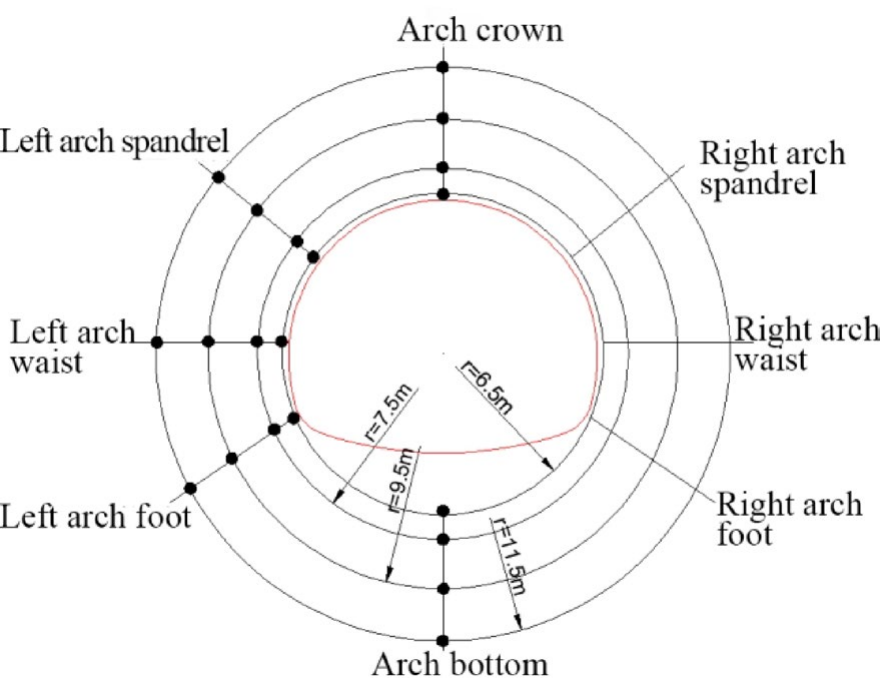
Arrangement of monitoring points around the tunnel perimeter.

**Fig. 15:**
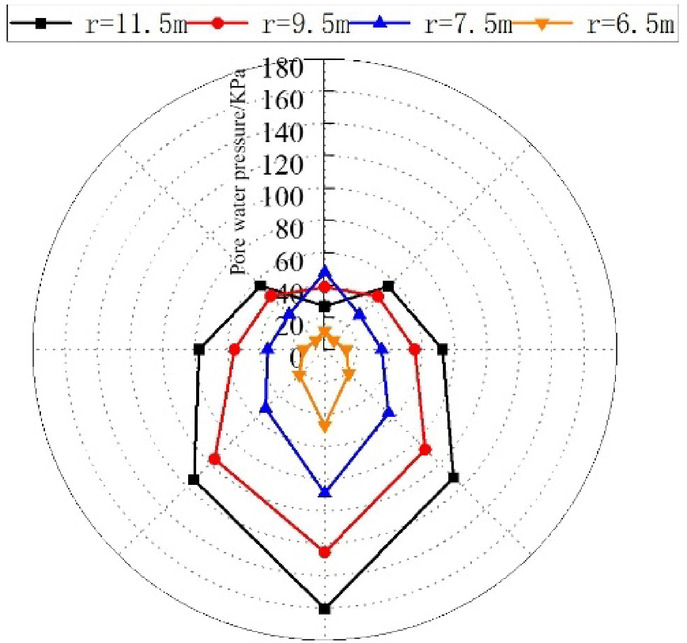
Variations in pore water pressure at different radial distances.

Within varying radial ranges surrounding the tunnel perimeter, the pore water pressure values at various control points were extracted (arranged as depicted in Fig. 14). As a result, the variation law of pore water pressure with the distance from the tunnel center as shown in Fig. 15 is obtained.

Figures 14 and 15 illustrate that when the groundwater table is 10 m above the arch crown and is defined as a constant water level, the order of pore water pressure magnitude at various measurement points in the tunnel is: arch bottom > arch foot > arch waist > arch shoulder > arch crown. Notably, the variation pattern of pore water pressure at the arch crown differs from other parts, increasing with the distance from the tunnel periphery within the pipe-arch reinforcement zone, and decreasing as the distance from the tunnel periphery increases above the reinforcement zone.

**Fig. 16:**
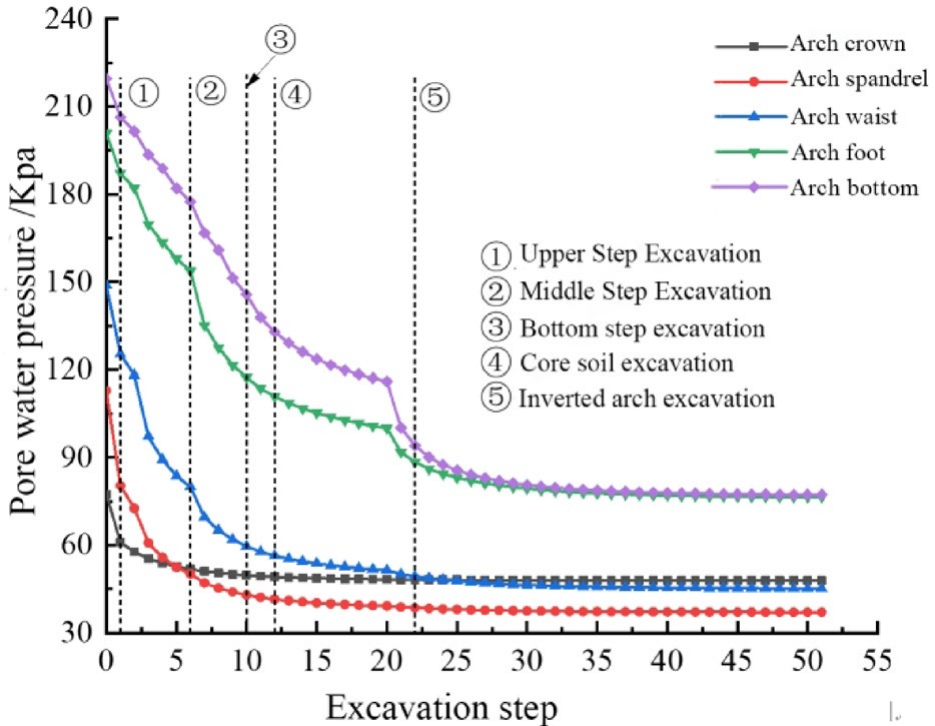
Evolution of pore water pressure across construction stages.

After the completion of tunnel excavation, the pore water pressure values at the arch crown, shoulder, waist, foot, and bottom within a range of r = 7.5 m were extracted and analyzed, resulting in the curves showing the variation of pore water pressure with construction steps as depicted in Fig. 16. It can be observed from Fig. 16 that the pore water pressure at the arch crown decreased from 77.4 kPa to 55.4 kPa after the upper bench excavation and then gradually decreased following the middle bench excavation, eventually stabilizing around 47.9 kPa. The pore water pressure at the arch waist, foot, and bottom exhibited a rapid decrease from after the upper bench excavation to before the inverted arch excavation, ultimately stabilizing at 45.2 kPa, 76.5 kPa, and 77.3 kPa, respectively.

After the upper and middle bench excavations, the seepage nodes at the arch crown and shoulder were activated, leading to a rapid change in pore water pressure in these areas. Following the lower bench excavation, the seepage boundary at the arch foot was activated, causing a swift change in pore water pressure in that region. After the inverted arch excavation, groundwater concentrated towards the arch bottom, resulting in a significant seepage gradient and consequently higher pore water pressures at the arch bottom and foot.

#### Comparative analysis of the surrounding rock displacement field

**Fig. 17:**
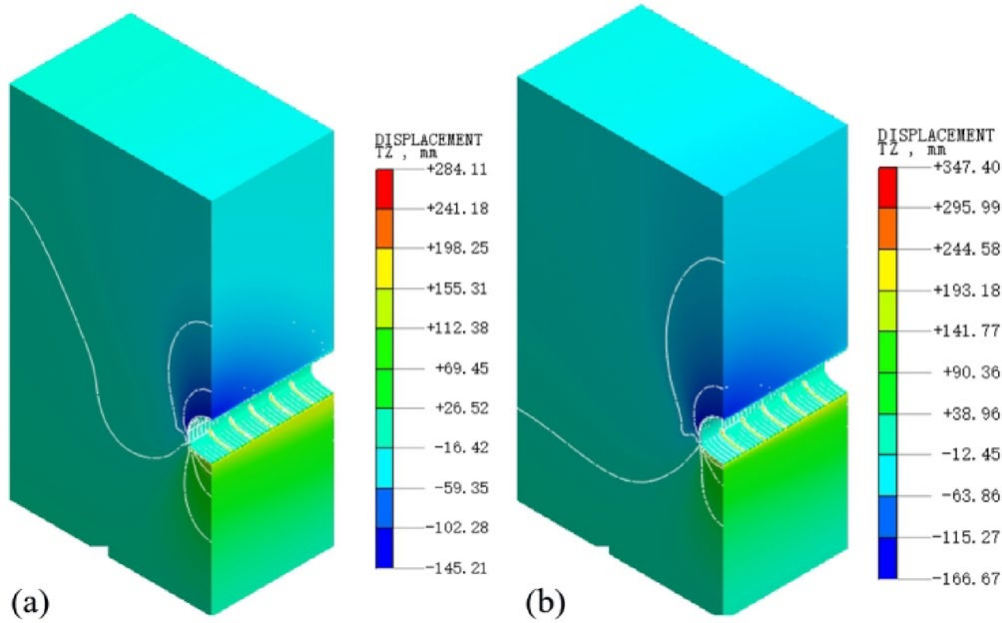
Contour maps of longitudinal vertical displacement under various working conditions: (**a**) without considering water migration; (**b**) water migration considered.

Figure 17a,b present the vertical displacement contour maps of the surrounding rock along the central axis of the tunnel under two different conditions (without considering water migration and with water migration considered).

**Fig. 18:**
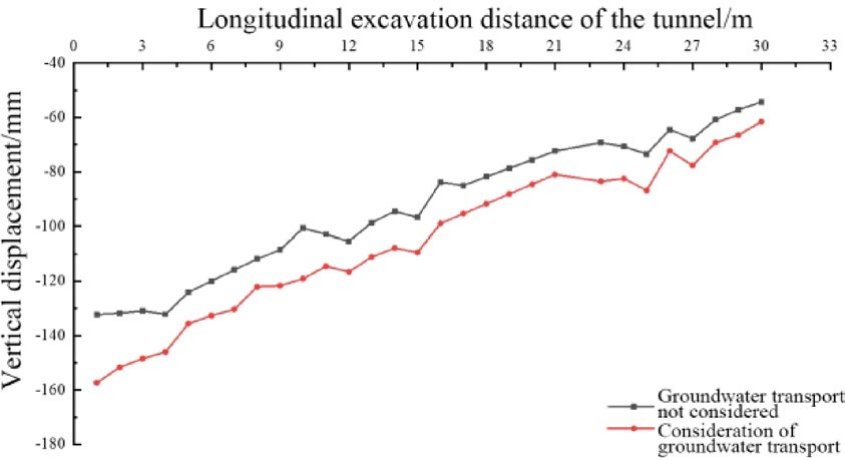
Curves of longitudinal vault displacement under different working conditions.

As can be seen from Fig. 17, the settlement patterns of the arch crown under both conditions are relatively similar. Numerical extraction was performed every 1 m along the tunnel excavation direction at the position of the arch crown, yielding the vertical displacements of various measurement points after the completion of tunnel excavation, as depicted in Fig. 18.

From Fig. 18, it is observed that under both conditions, the vertical displacement at the starting point of the tunnel model is relatively large, while the displacement at the endpoint is comparatively small. Due to the construction procedure settings, the secondary lining is applied directly after the excavation of the inverted arch, which leads to the control of the surrounding rock deformation. Consequently, the settlement of the arch crown exhibits a decreasing trend from the starting point to the endpoint. The settlement of the arch crown along the central axis of the tunnel increases when considering water migration, with the increment over the entire length being relatively uniform, ranging from 7.1 to 25 mm.

#### Comparative analysis of the stress field in surrounding rock

**Fig. 19:**
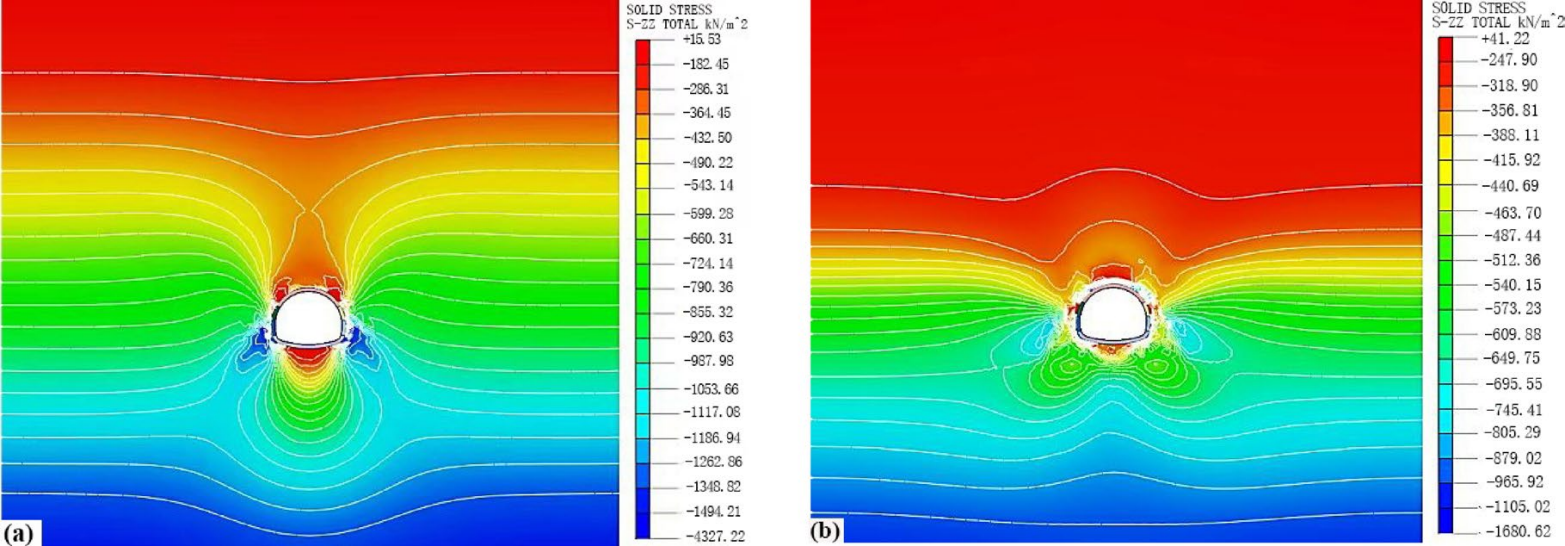
Stress contour maps of surrounding rock in the tunnel considering water migration: (**a**) Vertical stress; (**b**) Horizontal stress.

Figure 19 illustrates the stress contour maps after tunnel excavation considering water migration. It can be observed that compared to the scenario without water migration, the overall distribution of the surrounding rock stress has a larger influence range after the construction is completed. Under the influence of water migration, the variation of vertical effective stress is primarily at the crown and the foot of the arch, while the horizontal effective stress is mainly affected at the arch foot. Initially, seepage above the tunnel weakens the surrounding rock, reducing its load-bearing capacity and increasing the range of plastic deformation, resulting in a decrease in the stress in this area. Subsequently, the seepage causes the pore water pressure field to form a funnel shape, directing water flow toward the arch foot and the arch bottom. Under the three-step and seven-bench excavation method, the arch foot and bottom become the main seepage areas, leading to a dense contour line of pore water pressure and a significant increase in hydraulic gradient, causing a substantial increase in stress.

By extracting stress data from key points around the tunnel periphery, the comparative data of horizontal and vertical effective stress of the surrounding rock under two conditions are obtained as shown in Table 6.

**Table 6:** Comparison of horizontal and vertical effective stress at monitoring points.

Monitoring site	Without considering water migration/kPa			Considering water migration/kPa		
	Initial	Completion of excavation	Difference	Initial	Completion of excavation	Difference
Arch crown	− 394.7(− 701.3)	− 343.1(− 387.3)	− 51.7(− 314.0)	− 344.6(− 612.2)	− 337.6(− 387.1)	− 7.0(− 224.1)
Arch shoulder	− 400.6(− 712.0)	− 440.2(− 370.5)	39.6(− 341.5)	− 347.5(− 617.8)	− 441.5(− 321.8)	94.0(− 296.0)
Arch waist	− 442.2(− 786.6)	− 283.1(− 619.3)	− 159.1(− 167.3)	− 368.1(− 655.8)	− 271.5(− 586.9)	− 97.4(− 68.9)
Arch foot	− 464.5(− 825.4)	− 217.1(− 482.0)	− 247.4(− 343.4)	− 380.3(− 675.7)	− 187.7(− 405.1)	− 192.6(− 270.6)
Arch bottom	− 483.8(− 860.1)	− 291.3(− 54.8)	− 192.5(− 805.3)	− 389.9(− 693.6)	− 285.0(− 35.1)	− 104.9(− 658.5)

The values in parentheses represent vertical effective stress. Negative values in the difference column indicate decreases, while positive values indicate increases.

The data in Table 6 indicate that: (1) when considering water migration, the magnitude of horizontal effective stress decreases at the crown, waist, foot, and bottom of the arch, with the largest decrease at the arch foot, while it increases at the shoulder and wall of the arch; (2) the vertical effective stress at all points shows a decreasing trend. The reason is that when considering water migration, according to the principle of effective stress, the presence of pore water pressure reduces the vertical effective stress, and the greater the pore water pressure, the more pronounced the reduction in vertical effective stress.

#### Comparative analysis of stress on support structures

**Fig. 20:**
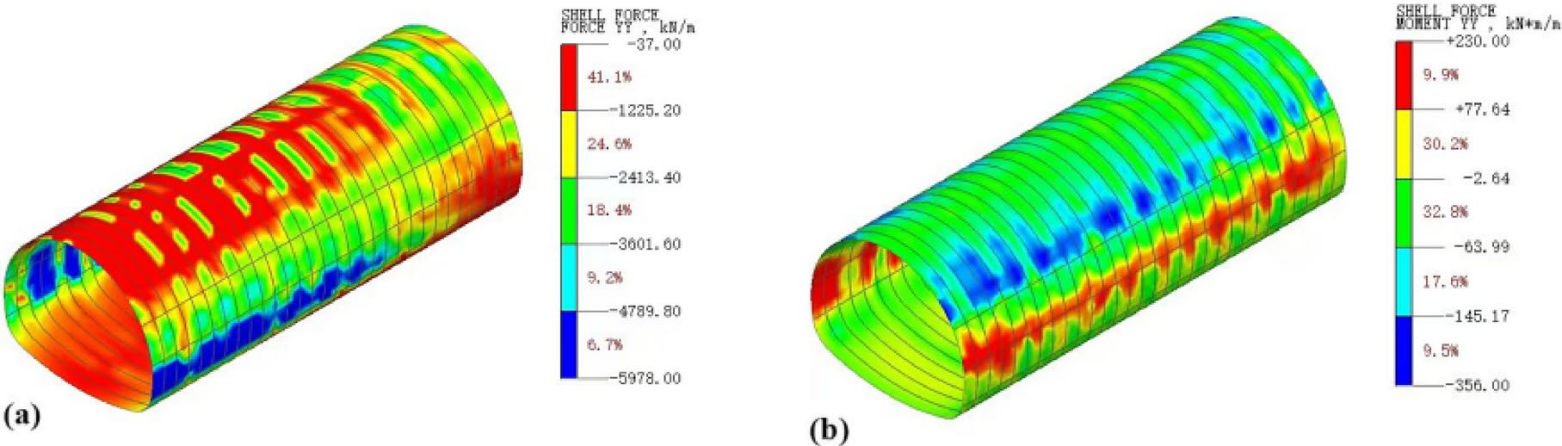
Contour maps of axial force and bending moment of support structure considering water migration: (**a**) Axial force; (**b**) Bending moment.

**Fig. 21:**
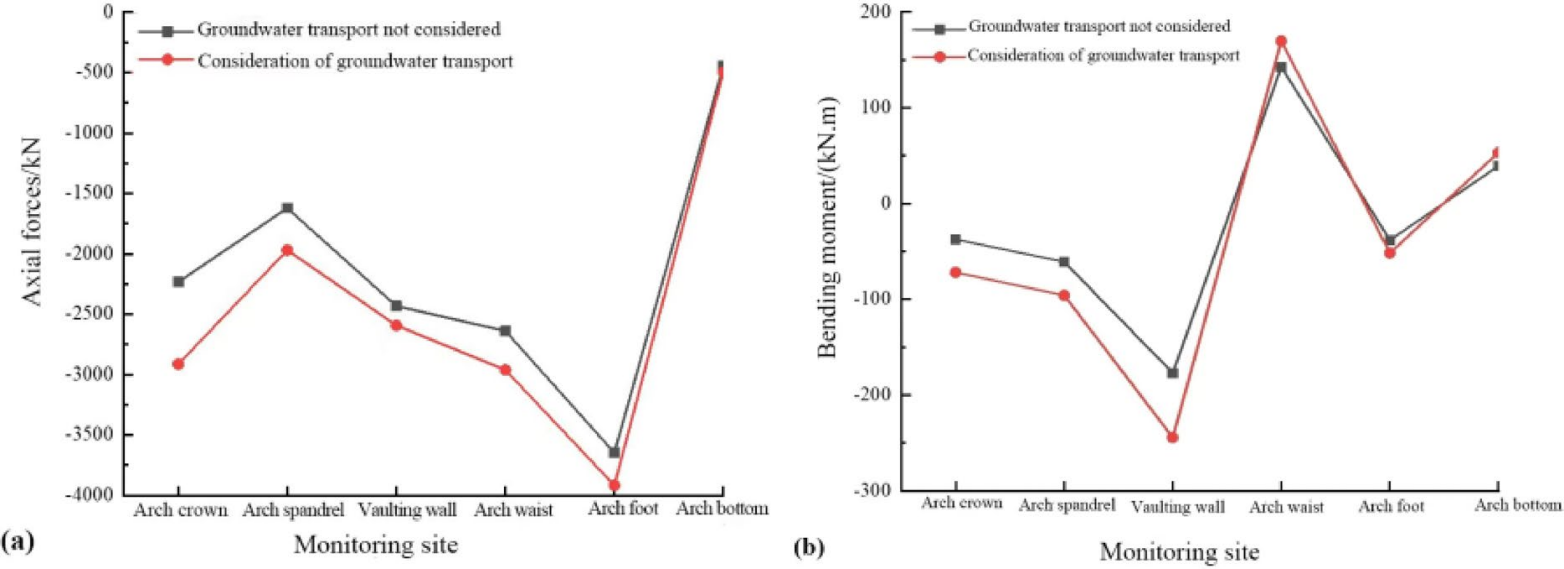
Characteristics of axial force and bending moment variations of support structure under different working conditions: (**a**) Axial force; (**b**) Bending moment.

The simulation results yielded the contour maps of axial force and bending moment of the support structure as shown in Fig. 20. Through the extraction and analysis of monitoring data, the characteristic curves of axial force and bending moment variations of the support structure under different working conditions were obtained, as depicted in Fig. 21.

Figures 20 and 21 reveal that the axial forces at the arch feet are substantial and the bending moments on both sides of the tunnel are notably high under both conditions. With the consideration of water migration, there is a significant increase in both the axial forces and bending moments in the initial support structure, reaching maximum percentage increases of 30.4% and 92.1%, respectively, at the arch crown. These findings underscore the pronounced effect of water migration on the stress characteristics at the crown.

## Discussion

1. For the shallow-buried monitoring section of this tunnel, the characteristics of pore water pressure variation are as follows: after the completion of the support structure, there is a slight increase in pore water pressure at both the arch foot and the crown of the monitoring section. In the crown area, pore water pressure increases with increasing distance from the tunnel periphery within the pipe-arch reinforcement zone, but exhibits a negative correlation beyond this area. This phenomenon is attributed to the pipe-arch reinforcement zone established within a 120° range of the arch, where the low permeability coefficient significantly impedes groundwater migration, leading to a dense contour of pore water pressure within the reinforcement zone and consequently higher pore water pressure in the surrounding rock above.
2. When considering water migration, the overall pattern of surrounding rock deformation is similar to that without consideration, but there are differences in specific values: the maximum vertical displacement at the monitoring section increased by 17.2%, and the horizontal convergence at the arch waist increased by 52.3%. Particularly after the upper bench excavation, the stress in the surrounding rock at various measurement points changed significantly, with the influence of water migration making this change more pronounced. For the shallow-buried monitoring section of the tunnel, the average settlement at the crown is 129.3 mm, and the average horizontal convergence is 24.5 mm, accounting for about 20% of the crown settlement, indicating that the tunnel deformation is primarily dominated by vertical settlement.
3. Regarding the content of parameter inversion, in this paper, the inversion verification was only conducted using the monitoring data of a single typical section, and the sample size was extremely limited, which may not be able to represent the characteristics of the entire tunnel surrounding rock. Moreover, the influence of the interrelationships and weights among different monitoring data on the inversion results was not deeply explored, which may lead to certain deviations in the inversion results. Further research should be carried out on how to scientifically determine the weights of different monitoring data and optimize the inversion algorithm. More practical engineering cases can be incorporated to strengthen the comparative analysis with other similar studies (such as the application of the PSO-XG Boost algorithm in the inversion of surrounding rock parameters), and to explore the applicability and advantages and disadvantages of different inversion algorithms under different working conditions.
4. In this numerical simulation, the groundwater was assumed to be at a constant water level. However, in the actual engineering situation, with the continuous drainage during tunnel excavation, the groundwater level is constantly decreasing. The decrease in the groundwater level inevitably leads to the consolidation settlement of the strata, and the settlement laws within different strata still require further investigation. During the seepage calculation, it was assumed that the geotechnical body was completely saturated, ignoring the changes in the physical and mechanical parameters of the surrounding rock caused by the changes in the saturation degree of the surrounding rock under the influence of seepage. For the loess tunnel in the shallow-buried section with a relatively low groundwater level, the seepage effect of unsaturated soil under water migration should be further studied.

## Conclusion

1. When assessing the impact of water migration, pore water pressure increases outward from the excavation face, particularly in the crown area, where the pipe-arch reinforcement effect results in a funnel-shaped dense distribution of water pressure contour, significantly enhancing the pressure on the surrounding rock and exacerbating the deformation and failure of adjacent weak surrounding rock. This leads to more pronounced vertical settlement and arch bottom heave compared to scenarios without considering water migration. Consequently, each stage of tunnel excavation must closely monitor changes in pore water pressure to ensure construction safety.
2. Fluctuations in the groundwater table caused by water migration significantly affect the displacement and stress at the crown and shoulders. Tunnel deformation is primarily characterized by vertical displacement, with significant stress concentration at the crown and arch bottom. Both the deformation of surrounding rock follow a three-stage change pattern: rapid growth in the initial stage, slowing down after support, and stabilizing after closure. During construction, the upper bench excavation has the greatest impact on vertical effective stress, the middle bench excavation mainly affects the arch wall and waist, and the lower bench excavation significantly influences the arch foot.
3. Comparing the measured and numerically simulated results of the water-rich section of the shallow-buried tunnel, it is found that the latter is generally smaller. The reason is that the numerical simulation did not incorporate the weakening effect of seepage flow on the mechanical properties of the surrounding rock, as well as the actual impact of rock mass joints and fractures. Compared to simulations without considering water migration, the simulation analysis of tunnel excavation considering water migration more closely reflects actual working conditions, providing a more accurate reflection of the surrounding rock deformation and the load on support structures.

## Data Availability

All data generated or analyzed during this study are included in this published article. The data and materials used in this article are available upon request by the correspondence author.
